# Evaluating tannery wastewater treatment performance based on physicochemical and microbiological characteristics: An Ethiopian case study

**DOI:** 10.1002/wer.1364

**Published:** 2020-11-05

**Authors:** Tesfaye Admassu Abate, Adey F. Desta, Nancy G. Love

**Affiliations:** ^1^ Institute of Biotechnology Addis Ababa University Addis Ababa Ethiopia; ^2^ Molecular, Cellular and Microbial Biology Addis Ababa University Addis Ababa Ethiopia; ^3^ Department of Civil and Environmental Engineering University of Michigan Ann Arbor MI USA

**Keywords:** 16S rRNA, bacterial diversity, chromium, Illumina, industrial treatment, leather manufacturing, water quality indicators

## Abstract

Tanneries are an important industrial sector in Ethiopia; consequently, gaps in wastewater treatment process performance need to be identified as the country increases its emphasis on compliance. A case study was conducted to evaluate physicochemical and microbial water quality at a tannery near Addis Ababa. The treatment process was designed for the following: sulfide oxidation; biological oxygen demand reduction; and chromium removal. While some of Ethiopia's standards for industrial wastewater treatment were met through treatment, effluent COD, sulfide, total nitrogen, and total chromium guidelines were not. 16S rRNA gene analysis was used to evaluate the microbial community composition across the treatment train. The results show that common ruminant phyla were dominant throughout, with Firmicutes and Bacteroidetes comprising 77% to 82% relative abundance. The Firmicutes *Clostridium* increased consistently in relative abundance with treatment, comprising 39% to 61% of the total bacterial community in the effluent. Improved treatment is needed to meet environmental and public health goals.

**Practitioner Points:**

- Case Study of tannery wastewater treatment in Ethiopia shows ineffective treatment of chemical pollutants.
- Microbiological pollutants from tannery wastewater systems can introduce agents of importance to public health
- The microbiological composition of tannery influent, mixed liquor and effluent contains mostly four bacterial phyla lead by Firmicutes.
- Most pathogenic bacterial genera found in the tannery wastewater treatment system became a decreasing percentage of the total population.
- *Clostridium* comprises up to 61% of the effluent bacterial population and deserves further evaluation to better understand the consequences of its dominance.

## Graphical abstract

Chemical and microbiological characteristics across a tannery wastewater treatment system in Ethiopia

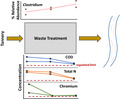

## Introduction

leather manufacturing is an industry with a long history in Ethiopia, which is the largest livestock producer in Africa and tenth largest in the world. The country's modern leather manufacturing method started in the 20th century. Today, more than 27 tanneries exist in the country that export semi‐finished and finished leather (Coppeaux et al., 2016), and a few more are under construction. The processing of leather is an export industry for Ethiopia, making up almost 6% of annual exports per year (around $127 million USD) (Simoes & Hidalgo, 2011). Ethiopia is among the poorest but fastest growing economies in the world and is evolving toward a market‐based economy. These changes are motivating the leather industry to produce high‐quality exports while meeting environmental and public health needs (UNCTAD, 2018); nevertheless, progress toward this end is slow given the country's economic constraints. Important to this end is as follows: understanding how the country's current approaches to industrial wastewater management are affecting both the environment and public health; determining what methods can be implemented to create best treatment practices; and creating useful water quality guidelines that can inform future regulations.

Although there is interest in moving toward “green,” chromium‐free leather manufacturing (UNCTAD, 2018; ELIA, 2019), the process of manufacturing leather from raw skins and hides at most tanneries in Ethiopia still uses a water‐ and chemical‐intensive chromium‐based tanning method that is performed using the following steps: beam house, tanning, retanning, and finishing (Gutterres, Benvenuti, Fontoura, & Ortiz‐Monsalve, 2015; Wosnie & Wondie, 2014). The beam house operation includes soaking skins and hides in lime, which facilitates the removal of hair, and then removing the residual lime by slowly reducing the pH with acid. Additionally, bating is conducted in the beam house using proteolytic enzymes to softening the skins and hides. Ultimately, the beam house produces the strongest COD wastewater with the greatest volume. The chromium tanning and retanning steps convert the collagen from the skins and hides into leather; it is this step that produces the most hazardous wastewater that contains a strong chromium residual (Goswami & Mazumder, 2014). Finishing includes stretching, buffing, and/or drying the tanned product. The wastewater treatment system that receives wastewater from all these steps aims to remove solid waste (e.g., grift, fibers), suspended and dissolved substances, and tannery‐specific pollutants, such as sulfides and chromium. It is also likely to receive factory wash waters that do not go through tannery processing.

Wastewater from the tanning industry is complex and, consequently, tannery wastewater treatment is difficult to afford in low‐income countries. Ultimately, tanneries are an important source of pollution in Ethiopian surface waters since most of the treated or semi‐treated tannery effluents are released to nearby rivers. Regulations were implemented for the effluent from tannery treatment systems (EEPA, 2003) and will necessitate the use of appropriate technologies to meet the standards. This is particularly important since most of the treated and semi‐treated tannery effluents are released to nearby rivers that serve as irrigation water for crops and animal agriculture. The effluent discharge limits for tannery wastewater treatment set by the Ethiopian Environmental Protection Authority (EEPA) target physicochemical parameters such as nutrients (e.g., phosphates, total nitrogen, sulfides), chromium (total and VI), and phenol. Currently, the treatment approach used at the tanneries around Ethiopia varies across locations, which is likely to result in varying performance.

Most of Ethiopia's tanneries are located in and around Addis Ababa and in the Oromia region, which is more water abundant than other parts of the country and can more easily support water‐intensive tanneries (Gebre & Van Rooijen, 2009). Many of these tanneries only have primary treatment ponds, but some tanneries have started incorporating secondary treatment. For instance, four tanneries in Addis Ababa have completed construction of secondary wastewater plants, and three additional tanneries have secondary treatment systems under construction (Hailemariam, 2019). Past studies from Ethiopia, India, and Brazil showed that a combination of biological and chemical treatment can produce improved effluent quality that meets environmental regulations based on chemical performance, although several studies point to high variability in effluent quality (Chowdhury, Mostafa, Biswas, Mandal, & Saha, 2015; Gutterres et al., 2015; Sugasini & Rajagopal, 2015; Terfie & Asfaw, 2015; Tsegaye & Kaba, 2017). The EEPA regulations target chemical pollution but do not include microbiological indicators, perhaps reflecting an assumption that microbiological contaminants are not likely to survive the harsh chemical methods used during the tannery process. However, microbiological indicators such as rumen‐originating pathogens like the Enterobacteriaceae, which includes *Clostridium*, or other pathogens that can be sources for zoonotic diseases (Kemunto et al., 2018; Knight & Riley, 2019), may be appropriate to include as their presence would suggest a failure by the factory to provide barriers to their environmental release. Both the microbial composition of tannery treatment processes and the microbial water quality of tannery effluents are poorly characterized. To address this gap, both culture‐dependent and culture‐independent (i.e., DNA‐based sequencing) methods can be used to advance our understanding of the microbial diversity in tannery treatment processes (Birtel, Walser, Pichon, Bürgmann, & Matthews, 2015; Rosselli et al., 2016) and the residual microorganisms left behind after treatment. One of the methods used to study water quality and the microbial ecology of wastewater treatment systems is based on Illumina sequencing of the 16S rRNA gene. This approach is enhanced by the growing size of reference databases and reduced sequencing costs (Barb et al., 2016; Derakhshani, Tun, & Khafipour, 2016). Importantly, as we learn more about the microbial ecology of these systems, relevant microbial indicators will be identified that help to characterize the effectiveness of treatment performance in reducing the risk of zoonotic pathogens.

The objective of this case study was to couple an investigation of both physicochemical and bacterial composition across a full‐scale tannery to better understand the treatment performance of the process and identify chemical and microbiological contaminants of concern. The outcomes of this case study can guide future regulatory needs that will best protect environmental and public health. We used an average‐sized tannery located within the populated city of Addis Ababa as our field site and employed conventional physicochemical water quality testing with Illumina sequencing of the 16S rRNA gene to quickly ascertain relevant microorganisms across the treatment process and effluent. We characterized the influence that tannery processing and wastewater treatment had on the effluent, its ability to meet existing regulated industrial treatment guidelines, and reflect on the potential impact of microbial agents that survive treatment.

## Materials and Methods

### Sample site and physicochemical analysis

**Figure 1:**
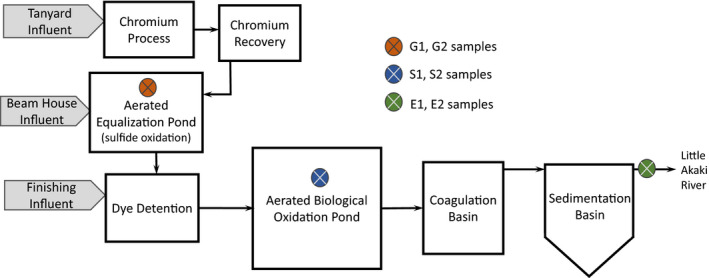
Schematic of full‐scale tannery wastewater treatment plant and associated sample locations.

This study was carried out at the tannery wastewater treatment plant located in the Akaki Kality, Sub City of Addis Ababa, Ethiopia. The tannery is located on the bank of the Little Akaki River (8^o^55′53″N, 38^o^45′29″E) to which tannery effluent is released. The tannery is a medium‐sized factory with an average processing capacity of 8,000 goat and sheep skins and 1,000 cattle hides per day (UNIDO, 2012). The tannery wastewater treatment plant is designed to remove phosphate, suspended solids, dye stuffs used in the leather finishing process, and chromium; nutrient removal was not fully evaluated in this study. The wastewater treatment plant influent is comprised of segregated inputs from the beam house, chrome, and dying operations. The treatment plant includes: aerated equalization; an aerated biological oxidation pond; and coagulation, flocculation, and sedimentation (Figure 1). The aerated equalization basin targets oxidation of sulfide. The biological treatment step targets oxidation of COD. Finally, the aerated mixed liquor from the oxidation pond is pumped to a small coagulation basin where chemical coagulants (alum and ionic polymers) are mixed in, flocculated and settled in the sedimentation basin for organics, phosphate and chromium removal. After sedimentation, the clarified effluent is discharged to the nearby Little Akaki River without disinfection or further tertiary treatment.

Samples were collected in sterile bottles during two sampling events (designated 1 and 2) that occurred ten days apart during the month of August (end of the wet season). Samples were collected at three distinct sites within each sample location and composited into one sample. Influent wastewater samples (denoted G) were taken from the equalization sulfur oxidation pond; the mixed liquor samples (denoted S) were taken from the aerated biological oxidation pond; and the final effluent samples (denoted E) were taken from the tannery effluent that was released from the sedimentation tank. Temperature, pH, and conductivity were measured on‐site during sampling with a digital portable pH meter that had separate probes for conductivity and pH/temperature (Thermo AP85 meter, Fisher Scientific, Singapore). Samples were transported to Addis Ababa University on ice in a cooler. Duplicate biomolecular samples were stored at −20°C, while all physicochemical samples were immediately processed or stabilized via freezing, according to established protocols. Analytes were measured by standard methods (Clesceri, Greenberg, & Eaton, 1999) or designated protocols as follows (by the given method number, where available): chemical oxygen demand (COD) by closed reflux (#5220); total dissolved solids (TDS) by a thermogravimetric method (#2540C); ammonia‐N by the Nessler method; sulfide by the methylene blue method (#4500 S^2‐^ D); sulfate by the turbidimetric method (#4500‐SO_4_^2‐^ E); total nitrogen by persulfate digestion (#4500N); and total chromium by inductively coupled plasma mass spectrometry (Agilent 7900). All analyses were conducted in triplicate, and reported standard deviations reflect triplicate analyses.

### DNA extraction and sequencing

All samples for biomolecular analysis were centrifuged at 2,000 *g*, and pellets were used to extract genomic bacteria DNA. DNA was extracted from duplicate samples per location using the Fast DNA^TM^ SPIN Kit for Soil (MP Biomedicals, Solon, OH, USA). Briefly, 300 mg of pellet and 978 μl of sodium phosphate buffer were added to a 2 ml microcentrifuge tube and vortexed for 15 s. A DNA stabilizing and solubilizing agent (MT buffer 122 μl) was added, and samples were homogenized by bead beating (BioSpec, Bartlesville, OK, USA) for 40 s. The homogenate was centrifuged at 14,000 *g* for 10 min, and the supernatant was transferred to a clean 2 ml microcentrifuge tube. Protein precipitating solution (250 μl) was added, shaken ten times by hand, incubated at room temperature for 10 min, and centrifuged at 14,000 *g* for 5 min to remove cell debris. Supernatant (800 μl) plus an equal volume of binding matrix was combined into a 2 ml tube and shaken gently by inverting five times. Finally, the DNA was eluted using 100 μl DNA elution solution and stored at −20°C until further use.

The integrity of the extracted DNA was visually verified using gel electrophoresis with 1% agarose and 1xTAE buffer. The quality and concentration of extracted DNA was verified with a Nano Drop 1,000 UV‐Visible spectrophotometer (ND‐1000 Thermo Fisher Technologies). Blank water was used during DNA extraction as a negative control, and no detectable DNA was recovered from the blank sample. DNA was submitted for sequencing at the University of Michigan's sequencing core (Medical College, Ann Arbor, Michigan, USA), where Illumina Miseq was performed with 2x250 paired‐end chemistry. An amplicon library was generated from the V4 region of the 16S rRNA gene after two step amplification of the DNA fragment using universal dual index primers 515F/806R (Mwaikono, Maina, Sebastian, Kapur, & Gwakisa, 2015; Saunders, Albertsen, Vollertsen, & Nielsen, 2015). The amplicons were indexed by barcodes and adaptors, which allow sequencing on the same flow cell and easier demultiplexing during sequence data analysis (Fouhy et al., 2015).

### Data analysis

The reads were analyzed using Mothur (version 1.36.1). Reads were filtered and de‐noised to remove low‐quality and ambiguous reads using the filter and screen codes. The two sets of reads were overlapped and combined to form contigs using the function make.contigs. Chimeric sequences were removed using the UCHIME algorithm embedded in Mothur by checking against chimera free data bases of 16S rRNA gene sequences following the sequence binning workflow. Sequence alignment was carried out using the Silva reference database (www.arb‐silva.de, version 123). Quality filtered sequences were assigned to taxonomic identities by reference database project (RDP) classifiers (Fadrosh et al., 2014), and sequences were clustered into OTUs at a 97% similarity threshold level using the UCLUST algorithm. OTU identification was performed using BLASTn (www.ncbi.org). Bacterial community richness was analyzed based on the number of OTUs obtained and using rarefaction analysis (Wu et al., 2015) after sub‐sampling (data not shown). Diversity indices (Shannon and inverse Simpson) were calculated using summary.single command. The number of OTUs in each sample was used to estimate diversity and evenness of bacteria community (Sinclair, Osman, Bertilsson, & Eiler, 2015). Principal coordinate analysis (PCoA) plots were generated from OTU data to assist with visualizing changes in diversity between samples. The observed core bacterial genera in each sample were presented in a heatmap using the clustvis package (Metsalu & Vilo, 2015). All sequences have been submitted to NCBI under submission numbers SAMN13738847 through SAMN13738852. Additional information about sequence data analysis is provided in the Appendix S1.

## Results and Discussion

### Tannery wastewater treatment underperformed

The physicochemical characterization of raw (G1 and G2), activated sludge (S1 and S2), and treated effluent (E1 and E2) samples from the tannery wastewater treatment plant is reported in Table 1. The water quality results from our study are presented as a range from the duplicate sampling days. Our results show much lower COD (2,100–4,000 mg/L) and sulfate (570–650 mg‐S/L) in the raw influent than found in the Modjo tannery (COD_avg_ = 12,500 mg/L and sulfate_avg_ = 800 mg‐S/L; Desta et al., 2014), and lower COD than the Dire tannery (COD_avg_ = 12,900 mg/L; Birhanie, Leta, & Khan, 2017). The total chromium concentration (13–37 mg/L) was similar or lower than these other studies (27–68 mg/L). Tannery wastewaters are highly variable (Gutterres et al., 2015), likely due to the type of leather tanning activity (chrome or vegetable‐based), the amount of hair, the cleanliness of hides and skins collected from different regions, and the complexity of the leather making process itself (Desta, Nzioki, Maina, & Stomeo, 2017; Saxena, Chandra, & Bharagava, 2016). Indeed, in our study we saw raw COD and ammonia‐N concentrations that were higher and total chromium concentrations lower in the G2 sample than G1, possibly reflecting different input stocks being processed by the tannery at the time of sampling.

**Table 1:** Physicochemical characteristics of the tannery wastewater treatment plant samples (mean ± STD) compared to the regulated limits or guidelines and other studies

	Influent (G), mixed liquor (S) and effluent (E) water quality results from this study						EEPA Discharge Limit^a^	Other studies	
Parameters	G_1_	S_1_	E_1_	G_2_	S_2_	E_2_		Raw influent	Treated effluent
pH	8.0 ± 0.1	8 ± 0.0	7.5 ± 0.1	8.3 ± 0.1	8.2 ± 0.1	7.9 ± 0.1	6.0–9.0	4.0–9.0^b^, 6.5–12.5^c^, 10.4 ± 0.3^d^, 8.2 ± 2.4^e^	7.7 ± 0.1^d^, 8.0 ± 0.1^e^
T (^o^C)	20.3 ± 0.5	18.7 ± 0.5	18.3 ± 0.5	20.0	18.0	17.3 ± 0.5	40	20.6 ± 2.34^d^, 20.3 ± 1.9^h^	18.0 ± 1.2^h^
Conductivity (mS/cm)	21.1 ± 0.1	19.4 ± 0.1	14.4 ± 0.1	25.5 ± 0.1	18.4 ± 0.2	13.5 ± 0.1	NL	15.5 ± 2.0^e^	8.2 ± 0.5^e^
COD (mg/L)	2,100 ± 30	2,040 ± 26	**1,070 ± 58**	3,990 ± 942	3,260 ± 72	**1,330 ± 67**	500	12,900 ± 6,900^c^ 12,500 ± 3,900^d^, 7,300 ± 540^e^, 1,760 ± 945^h^	395 ± 139^d^, 1,143 ± 262^e^, 338 ± 70^h^
TDS (mg/L)	4,310 ± 5	3,820 ± 9	2,220 ± 72	4,650 ± 11	4,190 ± 31	2,250 ± 133	NL	1,590 ± 508^h^	2,960 ± 223^h^
TSS (mg/L)	n/a	n/a	n/a	n/a	n/a	n/a	50	510–3,330^b^, 2,430 ± 515^c^, 1,160 ± 200^d^ 1,040 ± 438^h^	92 ± 11^d^, 90 ± 9^h^
Sulfide‐S (mg/L)	191 ± 4	99 ± 2	**58 ± 2**	212 ± 1	124 ± 2	**68 ± 3**	1	417 ± 131^c^, 56 ± 6^d^, 269 ± 76^e^, 92 ± 54^h^	4.9 ± 3.0^d^, 6.6 ± 3.8^e^, 0.3 ± 0.3^h^
Sulfate‐S (mg/L)	571 ± 13	464 ± 3	98 ± 1	646 ± 4	677 ± 3	669 ± 5	NL	800 ± 505^d^, 489 ± 71^e^	35 ± 61^d^, 433 ± 162^e^
Nitrate‐N (mg/L)	n/a	n/a	n/a	n/a	n/a	n/a	NL	124 ± 13^c^, 112 ± 24^e^, 11−18^f^	144 ± 35^e^, 9−11^f^
Ammonia‐N (mg/L)	32 ± 3	46 ± 0.8	1.4 ± 0.2	59 ± 2	54 ± 1	12 ± 1	30	24−762^b^, 36−127^f^, 34 + 23^h^	41 ± 22^e^, 32−35^f^, 79 ± 26^h^,
Total Nitrogen (mg/L)	289 ± 3	236 ± 2	**110 ± 2**	321 ± 3	294 ± 4	**118 ± 4**	60	545 ± 12^e^	220 ± 18^e^
Total Kjeldahl Nitrogen (mg/L)	n/a	n/a	n/a	n/a	n/a	n/a	n/a	265–12,900^g^	n/a
Total Cr (mg/L)	37 ± 0.9	4.6 ± 0.1	**4.8 ± 0.2**	13 ± 1	4.6 ± 1	**5.8 ± 0.3**	2	35.7 ± 8.6^c^, 27 ± 3^d^, 28 ± 5^e^, 15 ± 14^h^	7.7 ± 0.1^d^, 8.7 ± 7.2^e^, 0.6 ± 1.3^h^

Note

Bolded E1 and E2 values exceeded EEPA guidelines during this study. n/a, not available; NL, no limit set.

^a^ EEPA (2003);

^b^ Gutterres et al. (2015);

^c^ Birhanie et al. (2017);

^d^ Desta et al. (2017);

^e^ Alemu et al., (2019);

^f^ Sugasini and Rajagopal (2015);

^g^ Gutterres et al. (2015);

^h^ Tsegaye and Kaba (2017).

Removal, defined as the change between influent (G) and effluent (E) for each sampling event at the tannery treatment system, was 32% and 47% for conductivity; 49% and 67% for COD; 48% and 51% for TDS; 68% and 69% for sulfide; 79% and 96% for ammonia‐N; 63% and 64% for TN; and 54% and 87% for total chromium. These are moderate levels of removal in nearly all cases. Indeed, as shown in Table 1, the effluent from the tannery treatment system exceeded guidelines for COD, sulfide, total nitrogen, and total chromium in both samples. COD and total nitrogen loss occur mostly through chemically enhanced clarification. Presumably, the total nitrogen removed by coagulation reflects the organic fraction only. We do not have reliable nitrate measurements and cannot conclude that nitrification occurred; nevertheless, we do not find common aerobic ammonia or nitrite oxidizers in our sequencing data (Table S2), which suggest the wastewater treatment plant did not nitrify to a detectable extent. Other studies show that incomplete nitrification has occurred in Ethiopia's Modjo tannery (44% of effluent total N is nitrate) or a tannery in India (13%–18% of effluent total N is nitrate) (Sugasini & Rajagopal, 2015; Terfie & Asfaw, 2015). Nitrification is inhibited by both Cr(III) and Cr(VI) (Novotnik, Zuliani, Ščančar, & Milačič, 2014), as well as sulfide (Delgado Vela, Dick, & Love, 2018); these chemicals are found in the tannery effluent and could have contributed to limited nitrification. Furthermore, although TDS is not regulated, it was routinely high in the effluent and could have influenced the microbial ecology in the biological reactor and effluent. The chromium violation is of concern, given the potential toxicity of the metal (Saxena et al., 2016) and propensity to activate antibiotic resistance gene mobility between bacteria (Branco, Chung, VerÃ­ssimo, & Morais, 2005). Furthermore, alum is not a preferred coagulant for chromium (Johnson et al., 2008), and alternative coagulants should be considered. Overall, the water quality parameters exceeded the discharge limits from 1.5 to 63 times EEPA standards. Furthermore, variability in raw wastewater composition can make treatment more challenging; indeed, we see that the higher COD and ammonia‐N loads brought by G2 resulted in higher effluent COD and ammonia‐N concentrations (E2). Despite the lack of compliance with regulatory guidelines, most water quality parameters improved by the end of treatment during both sampling events; the exception to this was sulfate during the second sampling, which remained relatively constant. These results show that the treatment process used was either unable to or was not operated in a way that resulted in generation of an effluent with characteristics within EEPA guidelines. Furthermore, this confirms that modified treatment approaches are needed to improve effluent quality, and to protect public and human health from chemical pollutants of concern.

### Dominant bacterial phyla reflected rumen origins

**Figure 2:**
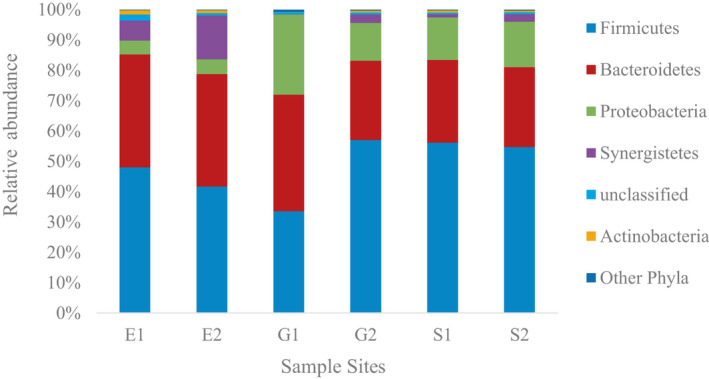
Composition and relative abundance of bacteria at the phylum level across all samples in the tannery wastewater treatment plant during the two sampling dates. Only phyla with relative abundance greater than or equal to 1% are shown.

Illumina sequence data were successfully annotated and characterized to produce bacterial community structure information from phylum to genus. The distribution of bacteria across the treatment system was dominated by four phyla: Firmicutes, Bacteroidetes, Proteobacteria, and Synergistetes (Figure 2 and Table S1). These phyla constituted over 98% of the total sequence reads. Firmicutes were the most abundant phylum in all sample points except G1, which was dominated by Bacteroidetes (38% versus 34%). The order of most to least average relative abundance among the influent and mixed liquor samples was (G, S): Firmicutes (45.3%, 55.4%), Bacteroidetes (32.2%, 26.8%), and Proteobacteria (19.5%, 14.5%) with Synergistetes a distant fourth (1.4%, 1.8%). The order of average relative abundance in effluent samples was different: Firmicutes (44.8%), Bacteroidetes (37.1%), Synergistetes (10.5%), and Proteobacteria (4.8%). In all sample locations, Firmicutes and Bacteroidetes are at least 77% of the total sequences, which show their dominance and persistence across the tannery wastewater treatment plant.

The dominance of Firmicutes and Bacteroidetes can be explained both because the tannery wastewater is exposed to fluids from ruminant and nonruminant animals, and possibly because of the prevalence of chromium. Firmicutes are mostly Gram‐positive, low G + C content anaerobic and facultative aerobic bacteria while Bacteroidetes are Gram‐negative, nonspore‐forming aerobic and anaerobic bacteria. Both phyla are known to ferment undigested carbohydrates and are often among the most dominant taxa in the rumen of bovine (Granja‐Salcedo et al., 2017; Jami & Mizrahi, 2012; Liu, Zhang, Zhang, Zhu, & Mao, 2016; Tapio et al., 2016), sheep (Tanca et al., 2017), and goat (Han et al., 2015; Liu et al., 2017; Wang et al., 2016), as well as the gastrointestinal tract of equine (Shepherd, Swecker, Jensen, & Ponder, 2011), rabbit (Monteils, Combes, & Godon, 2008), and human (Smith et al., 2019). Another study reported the high relative abundance of Firmicutes (46%) and Bacteroidetes (36%) in animal manure (Ozbayram, Ince, Ince, Harms, & Kleinsteuber, 2018). In addition to their role in mammalian guts, Firmicutes (especially *Clostridium* genus) were found by DNA‐based methods to be a dominant taxa in a chromium‐contaminated soil (Desai, Parikh, Vaishnav, Shouche, & Madamwar, 2009) and soils treated with chromium‐contaminated tannery sludges (Miranda et al., 2018), which suggest that strains exist within this taxa that are tolerant of chromium‐contaminated environments.

The next most predominant phyla were Synergistetes and Proteobacteria. The distinct prevalence of Synergistetes over Proteobacteria in the effluent was consistent across both sample dates, and suggests that the treatment process at the tannery influenced this selective shift. The dominant bacteria found in the tannery treatment system are different from what are typically found in hundreds of domestic wastewater treatment plant activated sludge samples collected globally, where Proteobacteria represent over ¾ of all bacterial taxa and would be expected to represent the majority of effluent taxa (Wu et al., 2019). Interestingly, the dominant phyla identified in this study were also reported in another study that used 16s rRNA gene clone libraries with samples from a pilot plant at the Modjo tannery in Ethiopia. That tannery employed a distinctly different treatment process (anaerobic/aerobic biological treatment followed by constructed wetlands; no coagulation step was used). In the Modjo study (Desta et al., 2014), Firmicutes and Proteobacteria dominated the last root zone samples from the wetlands, which are assumed to reflect the effluent, followed by Bacteroides and Cyanobacteria. *Synergistetes*, which are anaerobic bacteria, were also detected in the Modjo study. This shows that the nature of treatment can significantly influence the characteristics of the microbial communities present in the effluent from a tannery treatment plant. It also shows that Firmicutes is consistently present as an abundant phylum, independent of treatment method employed.

### OTU diversity varied with sample location and treatment approach

**Figure 3:**
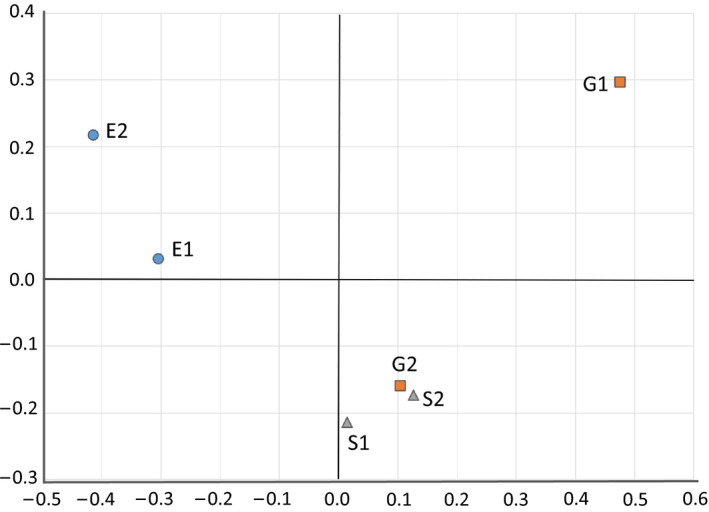
Principal coordinate analysis of the tannery wastewater treatment plant samples by location, using unweighted UniFrac distance as a measure of similarity between samples.

Mixed liquor (S) and effluent (E) sample OTUs reflected similar community structures within each sample type (i.e., S1 was similar to S2; E1 was similar to E2), while influent (G) sample OTUs were dissimilar between G1 and G2, as depicted by principal coordinate analysis (PCoA, Figure 3). The observed variability in the bacterial community structure in the influent samples may change due to changes in animal sources and tannery manufacturing practices, and is a common feature among tanneries (Amde, 2017; UNCTAD, 2018). The S and E samples each formed distinct clusters. This indicates two things: (i) samples within each S and E sample location were similar over time, and (ii) there is a sustained difference in microbial community structure between the S and E samples. The only treatment steps between S & E were coagulation, flocculation, and sedimentation. Therefore, the shift in microbial community composition was consistently influenced by this treatment, which removes insoluble or flocculant particles. Microbial strains detected in the effluent were either present in effluent suspended solids that originated in the mixed liquor (which in tanneries are expected to be below 50 mg/L), are able to exist in planktonic form, or are otherwise resistant to removal by coagulation and settling. The notion of an abundant planktonic fraction being present in tannery wastewater is consistent with the fact that rumen microbiomes include planktonic subpopulations that thrive in the rumen fluid (Cho et al., 2006). Finally, we see that the large community structure differences between G1 and G2 are not reflected in mixed liquor or effluent sample community structure changes. Therefore, at least over the ten‐day period reflected by samples in this study, the treatment system's microbial composition was resistant to influent changes. More sample points over time are needed to fully capture the variation of influent samples and how they impact mixed liquor and effluent community composition to fully evaluate the stability of the community.

**Figure 4:**
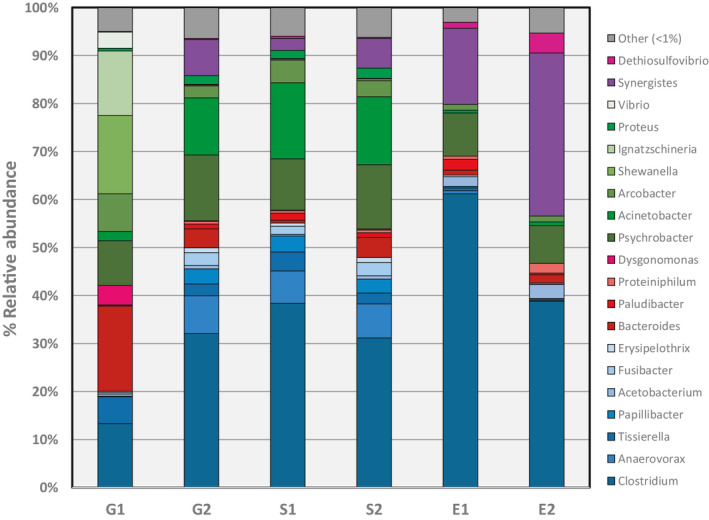
Relative abundance of classified Bacteria at the genus level for the three sampling locations across the tannery wastewater treatment plant during the two sampling events. Color‐coded bars are shaded in accordance with their phyla from Figure 2: Firmicutes blue shades; Bacteroidetes red shades; Proteobacteria green shades; and Synergistetes purple shades. Bacteria that were identified but exist at < 1% relative abundance are shaded gray and listed as other.

A comparison of the relative abundance among genera present in samples is presented in Figure 4. Variation in G1 and G2 is apparent based on OTU. G2 was dominated by: the Firmicutes *Clostridium* (32.1%), the Gammaproteobacteria *Psychrobacter* (13.6%) and *Acinetobacter* (11.9%), the Firmicutes *Anaerovorax* (7.9%), the Synergistetes *Synergistes* (7.6%), the Bacteroidetes *Bacteroides* (4.0%), and the Firmicutes *Papillibacter* (3.1%). In contrast, the four most dominant OTUs in G1 were similar in percent relative abundance and included: *Bacteroides* (17.8%), the Gammaproteobacteria *Shewanella* (16.4%) and *Ignatzschineria* (13.5%), and *Clostridium* (13.2%). Despite this variation between influent samples, the dominance of genus *Clostridium* and the class Gammaproteobacteria were reestablished in the mixed liquor and were consistent across both sample dates. The tannery mixed liquor bacterial structure is different from mixed liquors treating domestic wastewater, which are typically predominated by bacteria from the class Betaproteobacteria (Nascimento et al., 2018; Wu et al., 2019).

*Clostridium* was the most dominant genus in all sample locations (31.1%‐61.2%) except for G1 (13.2%), where it had comparable relative abundance to the other most dominant taxa. *Clostridium* was also dominant in mixed liquor samples from Modjo tannery (Desta et al., 2014). Effluent samples in the current study were also consistently dominated by *Clostridium*. Interestingly, although many strains within the phylum Firmicutes are biofilm formers, only a few biofilm forming strains of *Clostridium* have been studied from the many strains known to exist (Pantaléon, Bouttier, Soavelomandroso, Janoir, & Candela, 2014). *Clostridium* that feed on solid cellulosic substrates can form biofilms; however, they can be released as detached biomass or survive in the planktonic state in the absence of adequate substrate (Desvaux, 2005; Gelhaye, Petitdemange, & Gay, 1993). Furthermore, they are metabolically diverse (Xing, Guo, Tian, & Wu, 2011) and can live in either a growth‐supporting vegetative state that requires anaerobic conditions since they are strict (but not obligate) anaerobes, or as a nongrowing endospore that is highly resistant to disinfection and other hostile forms of treatment (Kiu & Hall, 2018; Mckew, Dumbrell, Taylor, Mcgenity, & Underwood, 2013). It is unclear how *Clostridium* persisted during the tannery process, across the tannery treatment plant, only to dominate in the system's effluent (either through growth, or by out‐surviving other strains that succumbed to coagulation–flocculation–sedimentation). Given the harshness of the chemical tannery process, it is reasonable to assume that surviving cells existed as endospores; however, whether they became vegetative cells in anaerobic niches of the treatment plant could not be discerned by our study and should be evaluated in a follow‐up investigation. Our results are consistent with the prior study by Desta et al. (2014), who used clone library analysis and showed that *Clostridium* was the most dominant genus in the last constructed wetland root zone sample collected from the pilot plant at the Modjo tannery.

Besides *Clostridium*, other genera within the Firmicutes appeared in the current study, but at much lower relative abundance across all samples. For example, the effluent samples showed *Acetobacterium* to be the most abundant Firmicutes after *Clostridium* (1.9% and 2.9% RA in E1 and E2, respectively); however, this genus did not appear above 1% relative abundance in either mixed liquor or influent samples. Instead, *Tissierella* was 4.4 and 2.2% RA in mixed liquor and 5.6 and 2.4% in influent samples. Similarly, *Anaerovorax* was 6.7 and 7.2% in mixed liquor, and 7.9% in G2 while it was 0.1% in G1. Finally, *Papillibacter* was 3.3 and 3.0% in mixed liquor, and 3.1% in G2 while it was also 0.1% in G1. Beyond the Firmicutes, *Synergistes* (Synergistetes) had the second largest relative abundance in effluent samples (15.8%, 33.9%), and *Psychrobacter* (Proteobacteria) was third at 9.0% and 7.9% RA. Consequently, the effluent became dominated by a few OTUs.

To evaluated changes in diversity across the treatment plant locations, alpha diversity was estimated across all samples (Table S3). Community richness, defined by number of OTUs characterized, was greatest in mixed liquor samples across both sample dates (1,059 and 1,008), which is not surprising. Interestingly, influent sample G1 that was structurally quite different from all other samples also had the next lowest richness (485 OTUs), and was quite different from G2 (977 OTUs). Effluent samples had the lowest richness, with 874 and 873 OTUs. Community diversity was evaluated by the Shannon diversity and inverse Shannon indices (Table S3). Both show that effluent samples were least diverse, and influent samples were most diverse although both influent and mixed liquor samples had comparable indices. The reduced diversity of the effluent is consistent with what is shown in Figure 4, due mostly to the dominance in the observed relative abundance of one (*Clostridium*) or two (*Clostridium* and *Synergistes*) OTUs. Finally, all samples showed low evenness (value ≤ 0.1), indicating that the communities are dominated by a relatively small number of OTUs. Indeed, only 20 OTUs had relative abundance measurements at or above 1% among 254 total OTUs characterized, and reflect 94% to 97% of the relative abundance among classified taxa. This outcome justifies focusing community analysis on a subset of taxa, which we define as those present at a relative abundance ≥ 1%.

Importantly, most of the Illumina sequence reads at the genus level were not annotated and presented as unclassified reads (Figure S1). A comparable high proportion of unclassified bacteria at the genus level was reported in the gastrointestinal tract of cattle (Kim et al., 2014), the feces of milk cows (Liu et al., 2016), and pond water samples (Qin et al., 2016). This reflects a current limitation of applying 16S rRNA‐based community analysis to understudied environments, such as tanneries, despite knowing the source of wastewater entering the treatment plant comes from processing of hides and skins. As more sequences from industrial treatment systems are uploaded into public databases, these limitations will be overcome.

### Tannery wastewater treatment enhances removal of most potential bacterial pathogens except *Clostridium*

**Figure 5:**
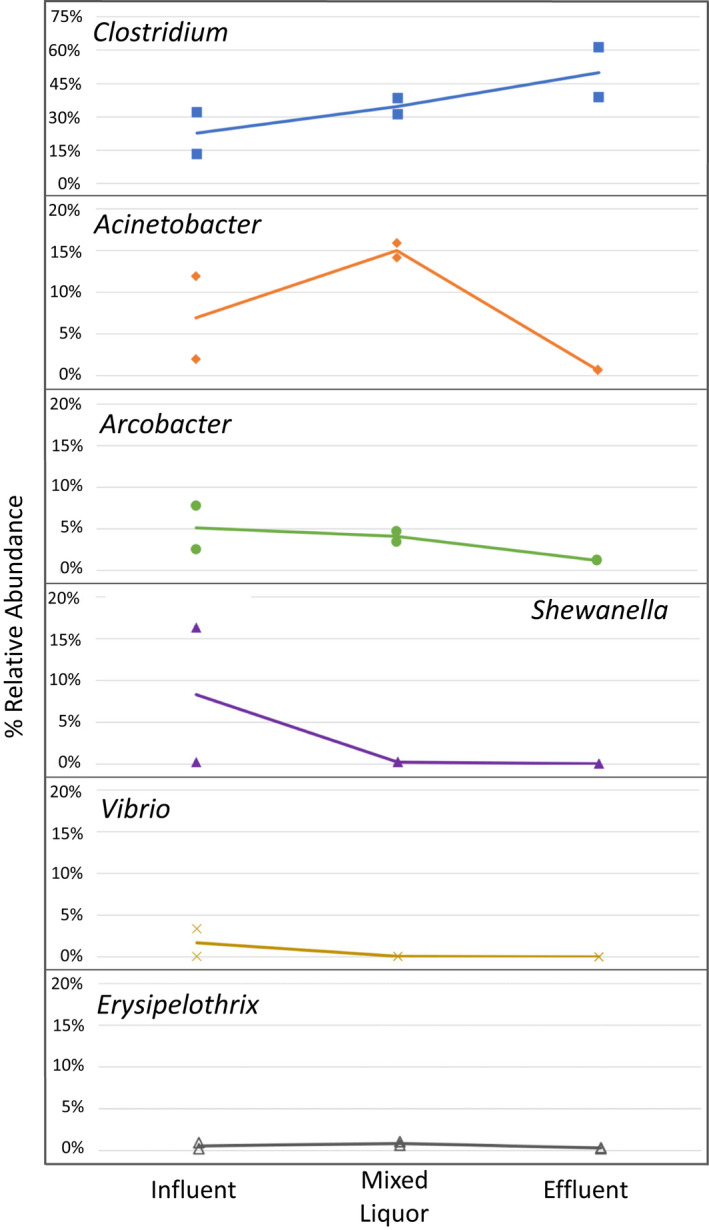
Changes in relative abundance of potential pathogenic bacterial genera across different sample points in the tannery. Symbols show the measured relative abundance in composited samples for each of two sampling dates, and the line graph shows the average values. Note that the scale for *Clostridium* is different from the others.

An important consideration of the bacteria found in the tannery treatment system is that six of the core (>1%) OTUs are of genera that include pathogens of importance to human health. These six genera are: *Clostridium*, *Acinetobacter*, *Arcobacter*, *Shewanella*, *Vibrio,* and *Erysipelothrix*. While our methods cannot discern if the taxa present are, indeed, pathogenic, we evaluated these genera to assess the effectiveness of the tannery treatment process to reduce their relative abundance in effluent samples. Our assumption is that a lower relative abundance translates into a lower risk of sending pathogenic strains into the receiving stream. As shown in Figure 5, the tannery was effective at reducing or maintaining the percent relative abundance between the influent and effluent for five of the six potential pathogenic genera. The largest change from influent to effluent among these five in at least one sample per location was *Acinetobacter* and *Shewanella*; we detected changes from 12% to 0.7% and 16% to 0.01%, respectively. Interestingly, in the case of *Acinetobacter*, the relative abundance peaked in the mixed liquor. *Arcobacteria*, *Vibrio,* and *Erysipelothrix* show continuous, albeit modest, reductions in relative abundance from influent through the aeration basin into the effluent. *Clostridium* was a notable exception relative to the other core potentially pathogenic bacterial genera. We see that it consistently increased in relative abundance across the treatment system to its highest levels in the effluent; next, we provide comments on the importance of this observation.

*Clostridium's* dominance in this study is relevant because several species within the genus (e.g., *C. difficile*, *C. perfringens*) are increasingly recognized as potential zoonotic pathogens of public health concern (Freeman et al., 2010; Knight & Riley, 2019; Rood & Cole, 1991). Importantly, zoonotic pathogenic infections are garnering increasing attention in East Africa, albeit without concomitant epidemiological studies (Kemunto et al., 2018). For this reason, it is important to pay attention to tannery discharges as potential point sources. While we cannot determine the species or physiological form of *Clostridium* present in effluent samples from the methods used in this study or if they were pathogenic, its ability to increase in relative abundance across treatment designed to remove particles suggests that planktonic forms of *Clostridium* may have been abundant in the effluent. *Clostridium* species can move between planktonic (the virulent form, if pathogenic) and sessile (more antibiotic resistant) forms, as needed (Crowther et al., 2014). Future studies that assess methods to enhance the treatment performance for tannery wastewater treatment plants should use culture‐dependent and culture‐independent approaches to monitor the fate of *Clostridium* across the treatment process, and determine whether the observations made here and at the Modjo tannery (Desta et al., 2017) are consistent with tannery treatment systems more generally. Notably, spore‐forming strains of *Clostridium* are known to be resistant to conventional disinfection (e.g., Kenters et al., 2017; LeChevallier & Au, 2004), which suggests that implementing such disinfection practices will not reduce their presence in the tannery effluent. Indeed, use of *Clostridium* as a water quality monitoring agent is gaining attention because, as a spore‐forming microorganism that is purely of fecal origin, it is more robust in the environment and resists die‐off that is typically seen with other, more common indicators (e.g., Stelma, 2018). The consequence of *Clostridium's* presence in Ethiopian tannery effluents and, presumably, receiving waters or their sediment deserves further attention, especially since many of these water sources are used routinely to irrigate food crops.

## Conclusion

Physicochemical analysis in the effluent to the Ethiopian tannery studied in this case study showed that several water quality parameters (COD, sulfide, total nitrogen, and total chromium) violated the country's industrial treatment standards during the time samples were collected. 16S rRNA gene‐based sequencing using the Illumina platform showed that the dominant bacteria found in the tannery influent wastewater were of the phyla typically found in the guts of ruminant animals, and persisted during treatment. In contrast, the chemical composition of the effluent varied more and appeared to reflect changes in chemical composition in the influent. Overall, the treatment approach used was insufficient to address expected variations in the influent, which can vary widely depending upon the type of animal skins being processed on any given day. Microbial community analysis showed that less than 8% of the OTUs comprised over 94% of bacterial phylotypes, reflecting low community richness. Furthermore, while the treatment process was able to reduce the relative abundance of most of the prominent genera that include pathogens, coagulation–flocculation–sedimentation resulted in an increase in the relative abundance of *Clostridium* in the effluent. An evaluation of which *Clostridium* species are present, in what forms (planktonic versus sessile, vegetative versus endospore), and their potential impact on public health is warranted.

## Conflict of Interest

The authors declare no conflict of interest.

## Authors’ Contributions

TAA collected and analyzed all samples. TAA and AFD conducted the primary data analysis. TAA and AFD did most of the data interpretation with significant input from NGL. TAA and NGL did most of the writing with significant input from AFD. All authors are responsible for the content of this paper.

## Supporting information

Table S1. Phyla from Illumina Analysis

Table S2. Genera from Illumina Analysis

Appendix S1

## Data Availability

Data beyond that provided in the paper and supplemental information are available from the corresponding author upon reasonable request.
