# Supplementary material for: Evaluating tannery wastewater treatment performance based on physicochemical and microbiological characteristics: An Ethiopian case study
Source: Water Environ Res. 2020 Nov 5;93(5):658–69. doi: 10.1002/wer.1364 (PMC8246915; doi:10.1002/wer.1364)
Supplement: Supplementary file 3 — Appendix S1 [file WER-93-658-s001.docx]

**Evaluating Tannery Wastewater Treatment Performance Based on Physicochemical and Microbiological Characteristics: an Ethiopian Case Study**

Tesfaye Admassu Abate, Adey Feleke. Desta, Nancy G. Love

**Supplemental Information**

***Supplemental Information about Sequence Data Analysis.*** The paired-end reads of the 16S rRNA gene were filtered and denoised to remove low quality and ambiguous reads following the online mothur SOP (http: //www.mothur. org/wiki/MiSeq_SOP) accessed in January 2016. The paired-end reads were assembled to make contigs, aligning sequences, trimming sequences, remove chimera by UCHIME algorithm embedded in Mothur by which low quality and non-bacterial sequence reads were removed following the sequence binning work flow. A total of 1,387,715 high quality 16S rRNA reads with a range of 179,994 to 308,999 and average of 231,286 reads were obtained from the six samples. From the total reads, nearly 99% of the reads were annotated at the phylum level, but the percentage of annotated taxa decreased to 83% at the class level, 67% at the order level, 56% at the family level and 39% at the genus level. Most of the sequence reads at the genus level were not annotated and presented as unclassified reads. At 97% similarity clustering, a total of 2,466 OTUs were generated across all samples and the OTUs were assigned into 28 phyla, 62 classes, 123 orders, 242 families and 441 genera.

**Table SI-1** Phyla from Illumina Analysis (separate file)

**Table SI-2** Genera from Illumina Analysis (separate file)

**Table SI-3.** **Diversity indices (at 97% similarity level) for the six samples collected from the tannery wastewater treatment plant.**

| **Diversity parameters** | **Samples** | | | | | |
| --- | --- | --- | --- | --- | --- | --- |
|  | **G1** | **S1** | **E1** | **G2** | **S2** | **E2** |
| **Reads** | 179,994 | 237,711 | 308,999 | 238,071 | 142,198 | 280,742 |
| **Number of OTUs** | 485 | 1059 | 874 | 977 | 1008 | 873 |
| **Shannon’s diversity (H’)** | 2.9 | 3.0 | 2.5 | 3.2 | 3.2 | 2.6 |
| **Effective number of species (e^H’^)** | 18.17 | 20.09 | 12.18 | 24.53 | 24.53 | 13.46 |
| **Evenness** | 0.1 | 0.08 | 0.05 | 0.09 | 0.1 | 0.05 |
| **Invsimpson** | 18.56 | 15.95 | 8.62 | 20.18 | 19.79 | 8.72 |

**Figure SI-1. Reads affiliated to classified (Orange line) and unclassified (blue bar) taxa in the tannery wastewater treatment plant.**
